# Parathyroid-hormone-related protein signaling mechanisms in lung carcinoma growth inhibition

**DOI:** 10.1186/s40064-015-1017-4

**Published:** 2015-06-17

**Authors:** Philippe R Montgrain, Jennifer Phun, Ryan Vander Werff, Rick A Quintana, Ariea J Davani, Randolph H Hastings

**Affiliations:** Medicine Service, Pulmonary and Critical Care Division, Department of Medicine, VA San Diego Healthcare System, UC San Diego, San Diego, USA; Research Service, Department of Biology, VA San Diego Healthcare System, UC San Diego, San Diego, USA; Research Service, VA San Diego Healthcare System, San Diego, USA; Anesthesiology Service, Department of Anesthesiology, VA Medical Center (125), VA San Diego Healthcare System, UC San Diego, 3350 La Jolla Village Dr., San Diego, CA 92161 USA

**Keywords:** Cell proliferation, Lung adenocarcinoma, Extracellular signal-related MAP kinases, Type 1 parathyroid hormone receptor

## Abstract

Parathyroid hormone-related protein (PTHrP) inhibits proliferation of several lung cancer cell lines, but the signaling mechanism has not been established. This study tested the hypotheses that growth inhibition is mediated through the PTHrP receptor, PTH1R, and that the process is modified by ERK activation. PTHrP-positive and negative clones of H1944 lung adenocarcinoma cells underwent stable PTH1R knockdown with lentiviral shRNA or transient transfection with ERK1 and ERK2 siRNA. Alternatively, cells were treated with 8-CPT cAMP, 8-CPT 2′-*O*-methyl cAMP, and *N*-6-phenyl cAMP analogs. H1944 cells expressing ectopic PTHrP showed 20–40% decrease in proliferation compared to the PTHrP-negative cells in the presence of normal levels of PTH1R (P < 0.01). PTH1R knockdown eliminated this difference and increased cell proliferation regardless of PTHrP status. The three cAMP analogs each inhibited proliferation over 5 days by 30–40%. ERK2 knockdown inhibited proliferation of PTHrP-positive cells alone and in combination with ERK1 knockdown. The growth inhibition mediated by cAMP analogs was unaffected by ERK1 knockdown. In conclusion, ectopic expression of PTHrP 1–87 inhibits H1944 cell proliferation. PTH1R knockdown blocks this effect and stimulates proliferation, indicating that the ligand exerts anti-mitogenic effects. cAMP, the second messenger for PTH1R also inhibits proliferation and activates ERK. PTHrP growth inhibition may be opposed by concomitant ERK activation.

## Background

The amino-terminal region of parathyroid hormone-related protein (PTHrP), residues 1–34, shares structural and functional features with the same region of parathyroid hormone (PTH). Thus, PTHrP 1–34 and PTH 1–34 activate the same G protein-coupled receptor, PTH1R. Furthermore, PTHrP causes the same effects with similar potency compared to PTH when the receptor is ligated (Abou-Samra et al. 1992). In lung cancer, amino PTHrP–PTH1R interactions are most frequently recognized in the syndrome of hypercalcemia of malignancy (Suva et al. 1987). Mid-molecule PTHrP peptides (residues 38–94) and carboxyl-terminal PTHrP peptides (residues 107–141) have biologic activities that are unique from the effects of PTHrP 1–34 (Orloff et al. 1994). For example, mid-molecule PTHrP fragments can regulate growth in breast cancer cells (Luparello et al. 2001), while the carboxyl domain affects function and proliferation in osteoclasts (Zheng et al. 1994). The non-amino effects of PTHrP are also presumed to result from binding to other cell-surface receptors, but such receptors have yet to be discovered (Orloff et al. 1994). In addition to cell surface actions, PTHrP can localize to the nucleus and can exert intracrine effects inside the cell (Gujral et al. 2001). Interestingly, the intracrine effects of PTHrP stimulate growth of vascular smooth muscle cells, in opposition to the anti-mitogenic receptor-mediated effects of PTHrP 1–34 (Stuart et al. 2000). Consequently, a variety of possible mechanisms must be considered to understand the effects of PTHrP in a given tissue or cell type.

PTHrP is expressed in two-thirds of human non-small cell lung carcinomas (NSCLC) (Hastings et al. 2006). In a previous study, we used an expression plasmid to evaluate the proliferative effects of amino PTHrP in H1944 lung adenocarcinoma cells and MV522 lung adenocarcinoma cells, two lines which possess PTH1R but normally do not express PTHrP (Hastings et al. 2009). A vector for PTHrP 1–87 was employed in this study because plasmids with shorter coding sequences function poorly for expressing the target protein (Ditmer et al. 1996). In both H1944 cells and MV522 cells, introduction of PTHrP resulted in slower increases in cell numbers and decreased incorporation of thymidine or BrdU into cellular DNA, but no apoptosis. This constellation of effects pointed toward changes in proliferation. The growth inhibition could be mediated by PTH1R or by a receptor for mid-molecule PTHrP, since PTHrP 1–87 includes both amino and mid-molecule sequences. Some investigators have suggested that PTHrP 1–87 can also cause intracrine signaling (Gujral et al. 2001), so this might be another mechanism regulating proliferation. PTHrP 1–87 transfection also stimulated phosphorylation of extracellular signal-regulated kinase (ERK). ERK signaling is generally associated with a proliferative response (Gollob et al. 2006), but prolonged activation of the pathway mediates cell cycle arrest in cells derived from a variety of benign and malignant tissues, including lung cancer (Wen-Sheng 2006; Clark et al. 2004; Sriuranpong et al. 2001). It is unknown what role ERK plays, if any, in PTHrP-dependent growth inhibition in lung cancer cells (Hastings et al. 2009).

The goal of this study was to establish the upstream mechanism through which PTHrP regulates lung cancer cell proliferation and to determine whether ERK is involved in the downstream signaling. We hypothesized that PTHrP activated ERK through PTH1R, leading to the inhibitory effects on proliferation. We used genetic approaches to alter PTH1R expression and ERK activity in H1944 cell clones that had been transfected with an expression plasmid for PTHrP 1–87 or with the empty vector to answer these questions.

## Methods

### Cell culture

NCI-H1944 human lung adenocarcinoma cells were obtained from the American Type Culture Collection (ATCC, Manassas, VA, USA). The cells were grown in a humidified incubator with 5% CO_2_/95% oxygen and in RPMI 1640 medium plus 8% fetal bovine serum supplemented with d-glucose, HEPES, sodium bicarbonate and sodium pyruvate. Prior to growth experiments, cells were incubated in serum-free medium with 1 mg/ml bovine serum albumin for 24-h to synchronize their growth cycle. Wild type H1944 cells do not make PTHrP. PTHrP-expressing H1944 cell clones produced by stable transfection make significant quantities of PTHrP, as determined by immunoassay (Hastings et al. 2009).

### Lentiviral shRNA knockdown of PTH1R

MISSION^®^ shRNA transduction-ready lentiviral particles directed against PTH1R promoter were obtained from Sigma-Aldrich Chemical (St. Louis, MO, USA). H1944 cells were seeded at 70–80% confluency in 48 well cell culture plates. The next day, the cells were transferred into fresh media containing 8 µl/ml hexadimethrine bromide and shRNA particles at 15 multiplicity of infection (MOI), which had been determined as optimum in preliminary trials. The shRNA particles were removed after another 24 h and replaced with fresh media containing 2 µg/ml of Puromycin. Stable clones were selected from colonies that survived exposure to a titrated lethal dose of puromycin antibiotic after several passages. Long-term inhibition of PTH1R expression in H1944 cells was assessed by immunoblotting. Control clones were stably transduced with non-target control (NTC) particles from Sigma.

### ERK siRNA transfections

H1944 PTHrP positive and negative cells were seeded at 2.5 × 10^5^ cells/well in 6-well plates to 40–50% confluency and transiently transfected with siRNA oligonucleotides that targeted ERK1 and ERK2 or non-silencing control sequences (sc-44205, sc-35335, and sc-37007, Santa Cruz Biotechnology, Santa Cruz, CA, USA). Each transfection used 20 pmol of siRNA oligomer in 500 µl serum-free RPMI mixed with 3 µl Lipofectamine 2000 (Invitrogen, Carlsbad, CA, USA). Cells were transfected for 48 h at 37°C in 1.5 ml of growth medium, then washed and returned to growth medium with 1 mg/ml bovine serum albumin substituted for serum for 24 h before beginning an experimental protocol. Efficiency of siRNA transfection was assessed by Western blot.

### Western blots

Protein was extracted from cell pellets in lysis buffer with phosphatase inhibitors (20 mM Tris–HCl, 150 mM NaCl, 1 mM Na_2_EDTA, 1 mM EGTA, 1% Triton X-100, 2.5 mM sodium pyrophosphate, 1 mM Na_3_VO_4_, 1 µM PMSF, 1× NaF and 1 µg/ml leupeptin). Complete lysis was ensured by exposing cells to a freeze–thaw cycle followed by sonication and vortexing. Lysates were cleared by centrifugation. Total protein concentrations were measured using a BCA assay (Pierce, Rockford, IL, USA). Wells in Criterion™ 4–12% Bis–Tris gels (Bio-Rad, Hercules, CA, USA) were loaded with 10 µg protein samples, separated by electrophoresis and transferred to polyvinylidene fluoride membranes. Membranes were blocked with 3% bovine serum albumin or 5% non-fat milk for 90 min at room temperature and probed with primary antibodies overnight at 4°C. Antibodies were directed against PTH1R (PRB-640P, Covance, San Diego, CA, USA), β-Tubulin, p-ERK (E-4) and ERK1 (K-23) (Santa Cruz Biotechnology), and p-Akt (serine 473) and total Akt. The epitope for the p-ERK antibody was a conserved sequence present in both ERK1 and ERK2 that contains a phosphorylated tyrosine, tyr 204 in ERK1. The ERK1 antibody recognizes a sequence within the XI subdomain in both phosphorylated and unphosphorylated forms of ERK1 and ERK2. After incubation with primary antibody, blots were then exposed to goat anti-mouse (sc-2031) or goat anti-rabbit (sc-2030) IgG-HRP conjugated secondary antibodies for 0.5–1 h at room temperature. Chemiluminescence was elicited by treatment with Amersham ECL Plus Western Blotting Detection Reagents (GE Healthcare) and recorded with the UVP BioSpectrum 410 imaging system (Upland, CA, USA). Band densities were analyzed with VisionWorksLS Analysis Software (UVP).

### PTHrP quantitative polymerase chain reaction (qPCR)

RNA was isolated from replicates of three cell culture wells each from H1944 cells, H358 lung adenocarcinoma cells and H441 lung adenocarcinoma cells with a mini kit (Qiagen, Valencia, CA, USA). The H358 cells and H441 cells are known to produce moderate levels of PTHrP. RNA was incubated with Moloney Murine Leukemia Virus reverse transcriptase (Invitrogen, Carlsbad, CA, USA) to produce cDNA for template. QPCR was performed on an ABI Prism 7000 real time PCR machine as previously described (Hastings et al. 2010) to amplify sequences from the PTHrP coding region and from β-actin. Relative abundance of PTHrP mRNA was calculated with β-actin as reference gene and H358 cells as the comparator by the Pfaffl method. Primers were forward 5′-CTGACACCTCCACAACGTCG-3′, reverse 5′-AGAATCCTGCAATATGTCCTTGG-3′ for PTHrP, and forward 5′-CCGAGGACTTTGATTGCACAT-3′ and reverse 5′-TTAGGATGGCAAGGGACTTCC-3′ for β-actin.

### MTS cell proliferation assay

Cells were plated at 1,500–2,500 cells/well in 96-well plates in growth media with FBS for proper cell adherence. In each experiment, cells were monitored by phase contrast microscopy to assess attachment and overall health. Assays in separate plates were stopped at 0–5 days after plating. The quantity of viable cells was assessed with a 3-(4,5-dimethylthiazol-2-yl)-5-(3-carboxymethoxyphenyl)-2-(4-sulfophenyl)-2*H*-tetrazolium (MTS) compound (Promega, Madison, WI, USA) after 1 h incubation at 37°C. MTS is bioreduced by metabolically active cells into a colored formazan product soluble in tissue culture media, which can be read at 490 nm absorbance. The absorbance is directly proportional to the number of living cells. In every experiment, a standard curve was constructed for each line relating absorbance to cell number. Results were analyzed in terms of cell number.

In some experiments, cells were treated at the start of the growth period with 1–200 µM 8-(4-chlorophenylthio)-adenosine-3′,5′-cyclic monophosphate (8-CPT-cAMP), 8-CPT-2′-*O*-methyl-cAMP (me-cAMP), or N-6-phenyl-cAMP (phe-cAMP). 8-CPT-cAMP is a non-selective agonist, while me-cAMP and phe-cAMP are selective for EPAC and protein kinase A (PKA), respectively.

## Results

### Role of PTH1R

#### PTH1R knockdown

**Figure 1:**
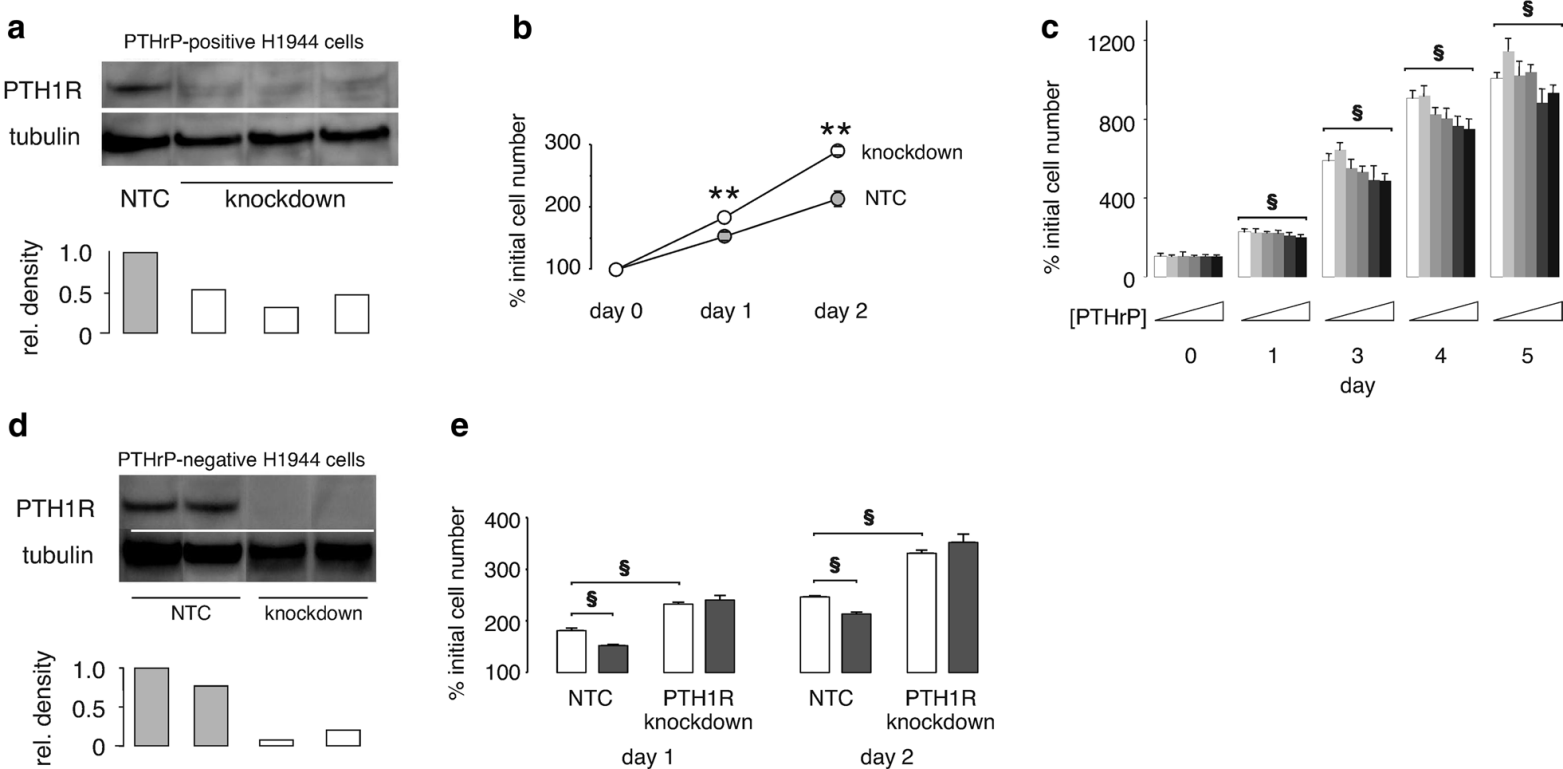
Effect of PTH1R knockdown on H1944 proliferation. **a** Stable transduction of PTHrP-positive cells with shRNA lentiviral particles directed against PTH1R (*open bars*) resulted in 55–70% knockdown of the protein compared to levels in cells transduced with NTC particles (*closed grey bar*). **b** The PTH1R knockdown clones demonstrated increased proliferation (*open circles*) measured by MTS assay compared to NTC clones (*closed circles*). N = 3 clones for each group. *Error bars* are smaller than the symbol for some points and may not be visible. **P < 0.01 vs. NTC cells at each time point. **c** Treatment with exogenous PTHrP 1–34 for 1–5 days decreased H1944 cell proliferation in a dose-dependent fashion. The *six columns* at each time point represent increasing PTHrP concentrations of 0, 10^−11^, 10^−10^, 10^−9^, 10^−8^, and 10^−7^ M. The reduction at the largest dose of 10^−7^ M was about 25%. Data are replicates of 8 cell wells per day and PTHrP concentration. Similar results were obtained in multiple independent experiments (^§^P < 0.001). **d** In PTHrP-null H1944 cells, stable shRNA transduction resulted in 75–90% knockdown of PTH1R (*open bars*) compared to levels in NTC control clones (*grey bars*). **e** When PTH1R was intact (NTC group), treatment with exogenous 100 nM PTHrP 1–34 (*closed grey bars*) inhibited proliferation of wild type H1944 cells by 25–50% compared to cells treated with the PTHrP vehicle (*open bars*) (^§^P < 0.001). However, exogenous PTHrP 1–34 had no effect on growth rate in clones after stable knockdown of PTH1R. Comparison of the open bars between PTH1R knockdown clones and NTC clones demonstrates that reduction in PTH1R expression increased proliferation even in the absence of exogenous PTHrP 1–34. ^§^P < 0.001.

Studies utilized three H1944 cell clones that make 600–700 pg PTHrP 1–34/µg cell protein/24 h as a result of stable transfection with the PTHrP 1–87 expression plasmid (Hastings et al. 2009). Cells from these clones were transduced with PTH1R shRNA particles or NTC particles to generate three PTHrP-positive, PTH1R knockdown clones and three clones with intact PTH1R. PTH1R levels in the knockdown clones were 30–45% of levels present in cells stably transduced with NTC shRNA (Figure 1a). By MTS assay, the PTH1R knockdown cells nearly tripled in number over a 2 days period, a 37 ± 1% faster growth rate when compared to the NTC-transfected cells, which only doubled in number over the same period (Figure 1b, P < 0.01). In comparison, wild type PTHrP-negative cells increased their numbers over 2 days by roughly 2.5-fold when PTH1R was intact and by about 3-fold when the receptor was knocked down (for example, see open bars at day 2 in Figure 1e). Thus, PTH1R knockdown reversed the growth inhibition induced by ectopic expression of PTHrP 1–87 and restored the rate of proliferation to levels close to those in PTHrP-null cells. These findings indicate that the presence of PTH1R suppresses cell replication.

Additional experiments evaluated the role of PTH1R in response to cell surface exposure to exogenous PTHrP. Exogenous PTHrP inhibited wild type H1944 cell growth in dose-dependent fashion (Figure 1c), as we have reported previously (Hastings et al. 2009). Wild type, PTHrP-negative H1944 cells underwent PTH1R knockdown to develop two independent knockdown clones along with two NTC clones. PTH1R knockdown clones differed from NTC clones in PTH1R level by 75–90% (Figure 1d). In the clones with intact PTH1R, treatment with the highest concentration of PTHrP 1–34 from the dose response experiment, 100 nM, decreased cell growth by 25 and 50% at days 1 and 2, respectively, compared to treatment with vehicle without PTHrP. However, exogenous PTHrP had no effect on the PTH1R knockdown clones (Figure 1e). Interestingly, PTH1R knockdown increased cell growth by roughly 60% compared to NTC controls in the absence of measurable PTHrP 1–34 (P < 0.001). This can be appreciated in Figure 1e by comparing the open bars between the knockdown and NTC groups. These data are consistent with two alternative explanations. Either the receptor possesses constitutive activity independent of ligand or that H1944 cells express levels of PTHrP 1–34 below the assay detection limit that still affect cell growth. qPCR assays indicate that H1944 cells contain quantifiable levels of PTHrP mRNA (Table 1), roughly 5–10% of the quantities in H358 cells and H441 cells. Given the presence of mRNA, H1944 cells could also be making low levels of PTHrP protein.

**Table 1:** PTHrP mRNA expression in human lung adenocarcinoma cell lines

Line	n	PTHrP CT	Δ CT	ΔΔ CT vs. H358	Relative abundance (95% CI)
		avg ± SE	PTHrP-β-actin		
H358	6	23.6 ± 0.6	8.8 ± 0.6	0.0 ± 0.1	1.00 (0.92–1.08)
H441	6	24.5 ± 0.1	9.9 ± 0.1	−1.0 ± 0.1	0.50 (0.45–0.54)
H1944	5	27.9 ± 0.3	13.0 ± 0.2	−4.2 ± 0.2	0.05 (0.04–0.07)

Values are avg ± SE unless otherwise indicated.

ΔCT values for β-actin ranged from 14.6 ± 0.6 to 15. 1 ± 0.2 for the three cell lines.

#### cAMP effect

**Figure 2:**
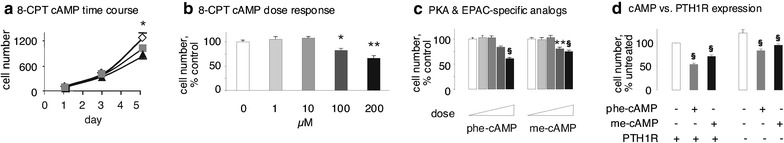
cAMP in PTH1R signaling and proliferation effects. **a** 8-CPT-cAMP attenuated the growth of H1944 cell number in a time- and dose-dependent fashion. *Open diamonds*, *grey squares* and *black triangles* represent cells left untreated or exposed to 100 and 200 µM 8-CPT-cAMP, respectively (n = 3 independent experiments, 6 replicates per experiment) *P < 0.05. **b** Increasing 8-CPT cAMP concentration caused progressively greater changes in cell number at day 5 with significant differences at 100 and 200 µM. *P < 0.05 and **P < 0.01 vs. untreated cells. **c** N-6-phenyl-cAMP (phe-cAMP), a cAMP analog targeting protein kinase A, and 8-CPT-2-*O*′-methyl-8-cAMP (me-cAMP), an EPAC-specific analog, both attenuated rate of increase in H1944 cell number over 5 days. Graph shows cell numbers at day 5. Concentrations range from 0 to 200 µM, as in panel **b**. **P < 0.01, ^§^P < 0.001 vs. untreated cells. **d** phe-cAMP and me-cAMP retained their growth inhibitory effects regardless of PTH1R expression. The figure shows results for PTH1R-expressing NTC clones (PTH1R+) and PTH1R knockdown clones (PTH1R−). Cell number is expressed relative to untreated PTH1R-positive cells. ^§^P < 0.001 vs. cells not treated with cAMP analog.

Since PTH1R stimulates cAMP as its second messenger in H1944 cells (Hastings et al. 2009) and BEN lung cancer cells (Hastings et al. 2004; Kukreja et al. 1988; Pizurki et al. 1988), we investigated the direct effects of cell-permeant cAMP compounds on H1944 cell growth. 8-CPT-cAMP, a non-selective analog, inhibited the rise in cell number in a time- and dose-dependent manner (Figure 2a, b). At a concentration of 200 µM, 8-CPT-cAMP reduced growth over 5 days by about 35%. Cells treated with 200 µM 8-CPT-cAMP for 4–6 days did not differ in their ability to exclude trypan blue compared to untreated cells, ensuring that the observed effect was due to growth inhibition, as opposed to cell death. Viability for treated and untreated cells was 92.6 ± 1.4 vs. 87.7 ± 3.9%, respectively after 3 days of treatment, and 92.0 ± 1.0 vs. 91.5 ± 1.0%, respectively after 5 days, with n = 6 replicates per group.

The growth inhibitory effects were not linked specifically to PKA or EPAC. N6-phe-cAMP, the PKA specific analog, and me-cAMP, the analog targeting EPAC, both inhibited H1944 cell growth (Figure 2c). At 200 µM, phe-cAMP reduced H1944 growth by 39 ± 2% (P < 0.001 vs. untreated cells), while the inhibition with 200 µM me-cAMP was 23 ± 3% (P < 0.001 vs. no treatment). By two-way ANOVA, growth inhibition depended on concentration (P < 0.0001) but did not vary between the two analogs (P = 0.37).

The cAMP analogs inhibited growth of the H1944 clones with PTH1R knockdown (Figure 2d) and the effect was similar in magnitude to the effect in cells with intact PTH1R expression. Thus, PTH1R knockdown prevented the anti-proliferative effect following treatment with PTHrP 1–34 (Figure 1b, e), but did not alter the inhibitory influence of cAMP (Figure 2d).

### Role of ERK activation

#### PTHrP effects on ERK

**Figure 3:**
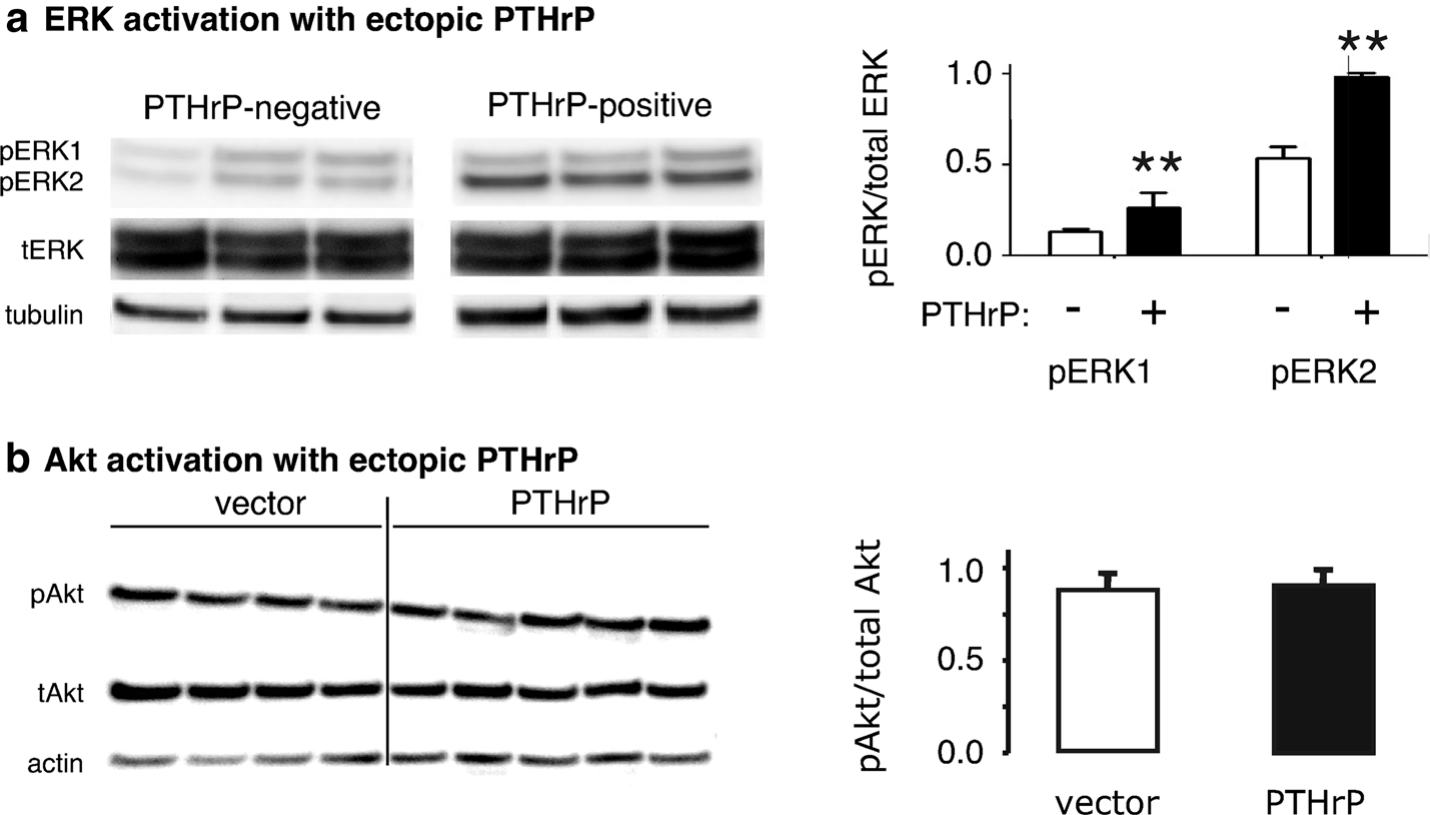
Kinase activation by PTHrP. **a** PTHrP-positive H1944 clones demonstrated increased levels of phosphorylated extracellular signal-regulated kinase (pERK) compared to the PTHrP-negative vector controls. Blots were performed multiple times with reproducible results. **P < 0.01 vs. vector control. **b** Ectopic PTHrP expression had no effect on Akt phosphorylation in H1944 cells. The figure shows immunoblots from 4 PTHrP-negative, vector-transfected H1944 cell clones and 5 independent PTHrP-positive clones.

Similar to our 2009 findings (Hastings et al. 2009), PTHrP-positive H1944 cells demonstrated pERK1 and pERK2 levels roughly twice as great as those in PTHrP-negative cells (Figure 3a). Total ERK protein expression was not altered significantly by PTHrP, based on immunoblotting. The level of pERK2 was roughly 4 times greater than the level of pERK1 in H1944 cells. In contrast to ERK, PTHrP expression did not activate Akt (Figure 3b).

#### ERK2 knockdown and PTHrP-mediated growth inhibition

**Figure 4:**
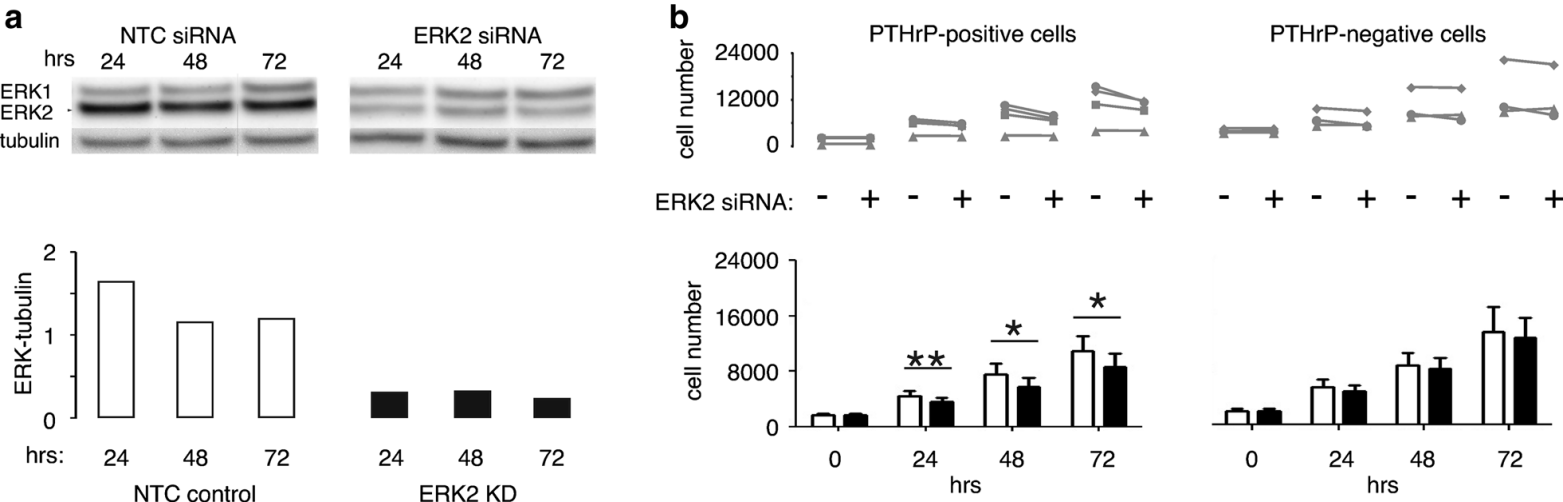
ERK2 knockdown and H1944 cell proliferation. **a** ERK2 siRNA (*closed bars*) knocked down expression of the target by 70–80% compared to NTC siRNA (*open bars*). The effect lasted at least 72 h. ERK1 was not affected by ERK2 knockdown. **b** H1944 cell proliferation was measured by MTS assay in 4 clones of PTHrP-positive cells and 3 clones of PTHrP-negative cells for 0–72 h after transient transfection with ERK2 siRNA or NTC oligonucleotides. The line segments in the *upper graph* connect points with and without ERK2 knockdown for the same clone and time point. ERK2 knockdown reduced proliferation over 72 h in 3 out of 4 PTHrP-positive H1944 cell clones. In contrast, ERK2 knockdown had no significant effect on proliferation in three PTHrP-negative clones. The *lower graph* presents averages ± standard errors, with *open bars* and *closed bars* representing transfection with NTC siRNA and ERK2 siRNA, respectively. Data were analyzed by paired t test comparing the effects of knockdown at a given time point between cells from the same clone. *P < 0.05, **P < 0.01 for NTC vs. ERK2 knockdown.

Transient transfection with ERK2 siRNA constructs resulted in 70–80% reduction in ERK2 expression in both PTHrP-positive and negative H1944 cells that persisted at least 72 h after treatment (Figure 4a). The ERK2 knockdown had no apparent effect on ERK1, and control, non-silencing oligonucleotides had no effect on either ERK species.

ERK2 knockdown reduced the rate of growth in MTS assays of three out of four independent PTHrP-expressing H1944 cell clones (Figure 4b). The reduction was significant, based on paired *t* test, at 24 h and later time points. At 72 h, proliferation was reduced by an average of 20.5 ± 3.4% in the three clones that responded to ERK2 knockdown. In contrast to the PTHrP-positive cells, knockdown of ERK2 in PTHrP-negative cells did not have a consistent or significant effect on cell proliferation (Figure 4b).

#### ERK1 knockdown and PTHrP-mediated growth inhibition

**Figure 5:**
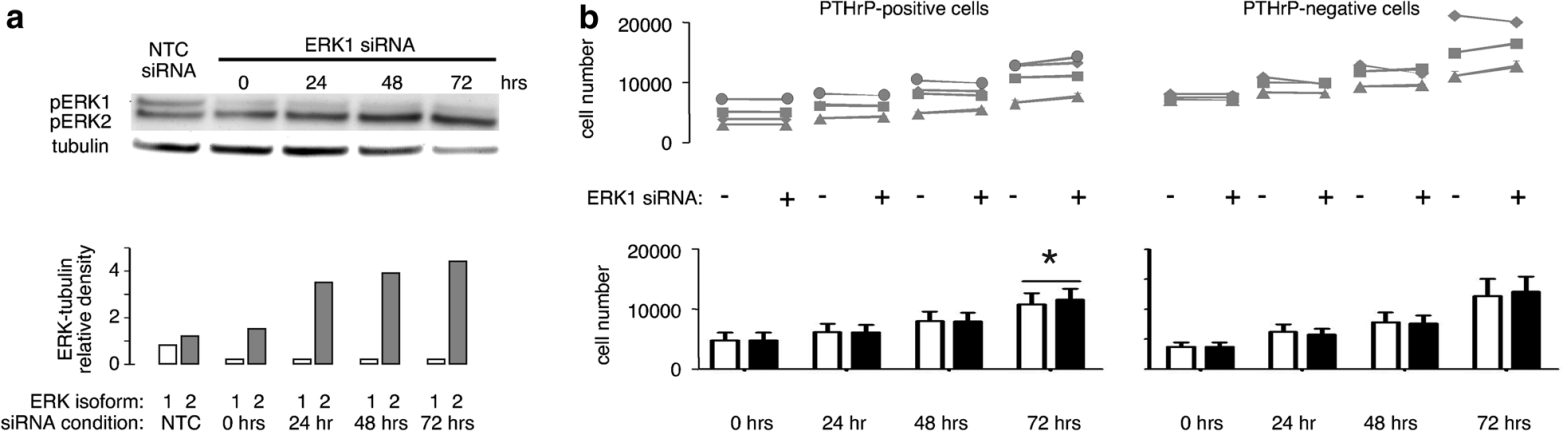
ERK1 knockdown and H1944 cell proliferation. **a** Transfection with ERK1 siRNA achieved 70–80% knockdown of ERK1 but also stimulated expression of ERK2. The *bottom panel* shows that the changes in ERK1 (*open bars*) and ERK2 (*grey bars*) lasted at least 72 h. **b** On the whole, transfection with ERK1 antisense constructs did not have a substantial effect on growth in four independent clones of PTHrP-positive cells or three clones of PTHrP negative cells. The formatting is similar to Figure 4, with line segments linking points from the same H1944 cell clone. ERK1 knockdown (*closed*, *black bars* in the *bottom graph*, *panel* **b**) resulted in a slight increase in cell number at 72 h compared to NTC siRNA treatment (*open bars in panel* **b**) in the PTHrP-positive cells. *P < 0.05 vs. control NTC siRNA by paired t test.

Transfection with ERK1 siRNA decreased ERK1 protein by 70–80% for 72 h (Figure 5a), but also led to increased expression of ERK2 protein. Levels of ERK2 increased by almost four-fold over the 3-day period.

Cells from PTHrP positive clones grew at the same rate after ERK1 knockdown as cells transfected with the control siRNA for the first 48 h, then showed a small 10% increase in cell number at 72 h (Figure 5b, left panel). ERK1 knockdown did not affect growth rate in PTHrP-negative H1944 clones (Figure 5b, right panel).

#### Combined ERK1/ERK2 knockdown

**Figure 6:**
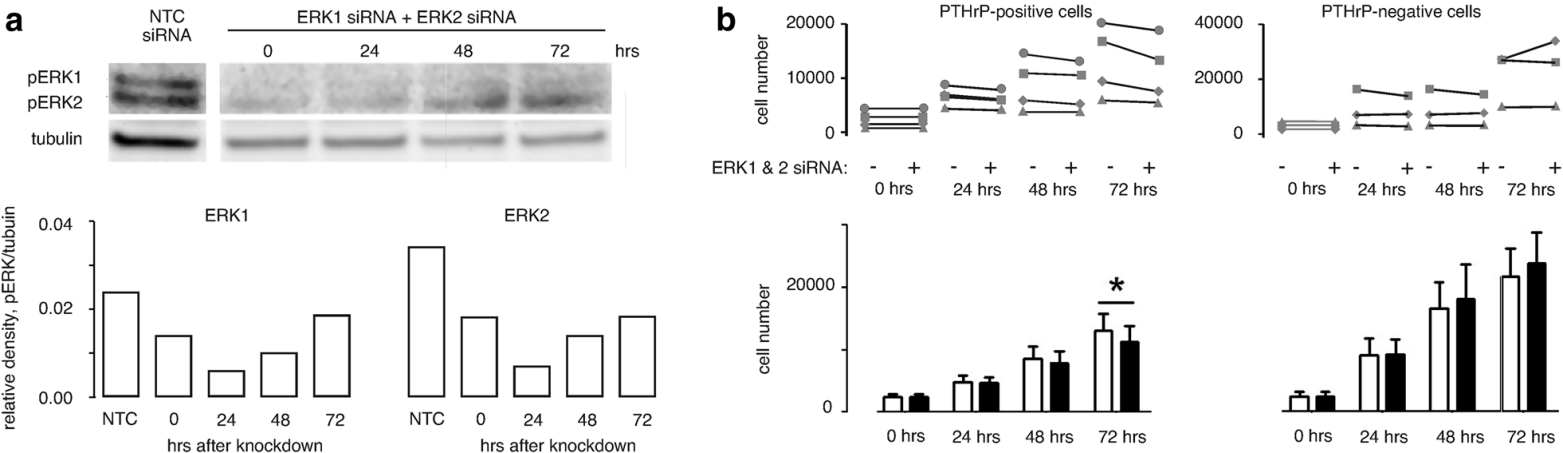
Combined ERK1 and ERK2 siRNA knockdown vs. proliferation. **a** H1944 cells were transfected with equal quantities of ERK1 siRNA and ERK2 siRNA for 48 h, and placed into fresh media for the indicated times up to an additional 72 h. The control consisted of NTC siRNA at the same total oligonucleotide concentration. Combined ERK1/2 knockdown led to 20–50% reduction in pERK1 levels and 50–75% reduction in pERK2 over the course of the 72 h. **b** The effect of combined ERK1/2 knockdown on H1944 cell proliferation was similar to that of ERK2 knockdown (Figure 4). Overall, the effects on growth were minimal except for a 13% decrease in proliferation of PTHrP-positive H1944 cells at 72 h. *P < 0.05 vs. control siRNA by paired t test.

To avoid the confounding influence of ERK2 upregulation, we combined the siRNA treatments to knock down both isoforms. ERK1 plus ERK2 knockdown decreased both pERK1 and pERK2 levels over 72 h. The reduction in pERK1 ranged from 20 to 50% knockdown, while pERK2 decreased by 50–75%, depending on the elapsed time after the transfection. Both ERK siRNAs gradually lost their knockdown efficiency over time, but pERK levels were still reduced compared to the control siRNA at 72 h (Figure 6a).

The dual ERK knockdown significantly decreased proliferation of PTHrP positive cells by 25% at 72 h post transfection but had no apparent effect at 24 and 48 h (Figure 6b). This finding was interesting because the extent of ERK2 knockdown at 72 h was less than it was at the 24 and 48 h time points (Figure 6a). Combined ERK1 and ERK2 siRNA knockdown did not cause any significant changes in the proliferation of PTHrP negative cells over 72 h.

#### Effect of cAMP after ERK1 knockdown

**Figure 7:**
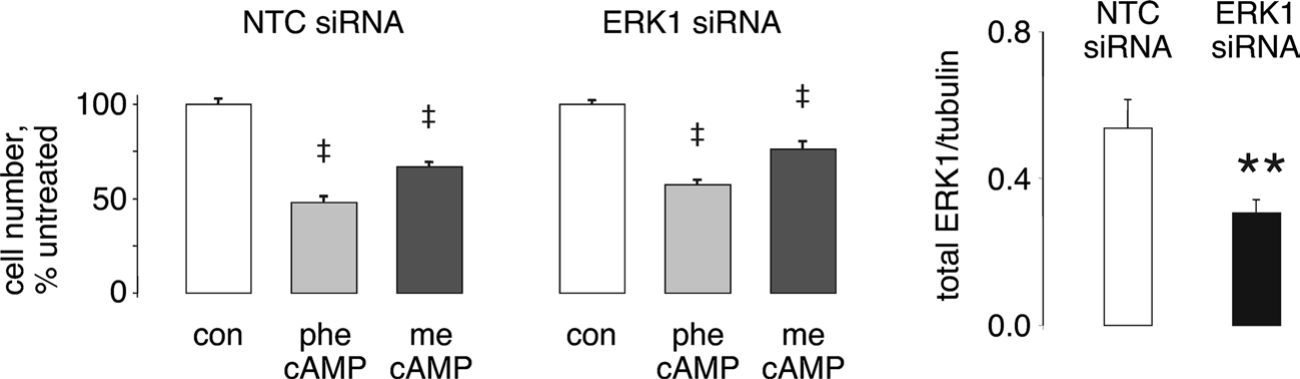
cAMP effects are independent of ERK. Wild type H1944 cells were transfected with oligonucleotides directed against ERK1 or against control, NTC sequences for 48 h as described in Figure 5. The transfected cells were replated and treated with media containing vehicle, 200 µM phe-cAMP or 200 µM me-cAMP and assayed for growth over 5 days. ERK1 knockdown had no effect on the growth inhibitory effects of the cAMP analogs. Inhibition for phe-cAMP amounted to 50–60% compared to vehicle and roughly 40% for me-cAMP. ^‡^P < 0.001. The *graph* on the *right* shows that the ERK1 siRNA reduced ERK1 expression by about 50%. **P < 0.01.

Since ERK1 knockdown stimulated proliferation, we investigated whether the reduction in ERK1 would alter cAMP-mediated growth inhibition. Treatment with cAMP analogs inhibited H1944 cell growth by 30–60%, even after 50% knockdown of ERK1. The anti-proliferative effect was similar to that observed when ERK1 levels were not manipulated (Figure 7), suggesting that ERK1 phosphorylation does not contribute in a major way to the suppression of cell division caused by cAMP.

## Discussion

In earlier work, we found that stable transfection of PTHrP 1–87 into PTHrP-negative lung adenocarcinoma cells inhibited proliferation (Hastings et al. 2009). The antimitogenic effects were associated with decreased expression of cyclin D2 and cyclin A2, increased expression of p27Kip and decreased activation of cdk2. In addition, ectopic PTHrP production led to phosphorylation of ERK. The current study investigated the signaling pathways involved in PTHrP-dependent growth inhibition.

PTH1R knockdown was employed to establish that the receptor had a role in limiting cell proliferation. Receptor knockdown abrogated the ability of exogenous PTHrP 1–34 to inhibit H1944 cell proliferation and it also eliminated the difference in growth rate between PTHrP-positive and vector-transfected H1944 cell clones. The demonstration that PTH1R is necessary for ectopic PTHrP 1–87 to reduce lung cancer cell growth excludes a significant growth inhibitory role for mid-molecule PTHrP domains, i.e. PTHrP 38–87 or PTHrP 67–87. These peptides do inhibit proliferation in breast cancer cell lines (Luparello et al. 2001).

Gujral and colleagues evaluated interleukin-8 expression in prostate cancer cells transfected with PTHrP 1–87 (Gujral et al. 2001), the same PTHrP form we studied. Ectopic PTHrP 1–87 significantly augmented IL-8 levels, but treatment with exogenous PTHrP 1–34 and PTHrP 67–86 peptides did not. They suggested that PTHrP 1–87 was acting through an intracrine pathway, although nuclear effects seem unlikely in this instance since the peptide is missing the PTHrP nuclear localization sequence found at residues 87–106 in the full length protein (Henderson et al. 1995). In any case, our study establishes that ectopic PTHrP 1–87 inhibits H1944 cell proliferation at an extracellular site, based on the requirement for PTH1R. Thus, the mechanism involves cell surface receptors, rather than intracrine pathways.

We have previously shown that PTH1R activation was coupled to Gs and generation of cAMP in H1944 cells and BEN lung cancer cells, rather than Gq pathways (Hastings et al. 1996, 2004, 2009). When cell-permeant cAMP analogs were tested in this study, they mimicked the effect of PTHrP in inhibiting H1944 cell growth. Furthermore, the cAMP effects persisted in the face of PTH1R knockdown, as would be expected if cAMP acted as the downstream second messenger for PTH1R. We attempted to delineate the signaling pathways by comparing the effects of two cAMP analogs that differed in selectivity for either EPAC or PKA. Me-cAMP and phe-cAMP both inhibited proliferation, suggesting either that EPAC and PKA independently inhibited cell proliferation or that they converged on a common inhibitory pathway.

PTHrP–PTH1R interactions have also been reported to cause cAMP-dependent growth arrest of vascular smooth muscle cells (Stuart et al. 2000). In smooth muscle cells, the effects involve an increase in p27kip1, inhibition of cyclin dependent kinase activity and reduction of Rb phosphorylation, similar to the effects of PTHrP in H1944 lung cancer cells (Hastings et al. 2009).

PTHrP and the cAMP analogs activated ERK in H1944 cells (Figure 3a; Figure 7b in Hastings et al. 2009). ERK activation could either contribute to growth inhibition or limit it depending on the direction of the ERK effect. The slower growth rate observed after either ERK2 knockdown or combined ERK1/ERK2 knockdown suggests that the overall effect of ERK phosphorylation is to stimulate proliferation. PTHrP-induced ERK activation also has a pro-proliferative effect in oral squamous carcinoma cells, osteoblasts, and colon cancer cells (Yamada et al. 2008; Datta et al. 2007; Martin et al. 2014). Our findings suggest that ERK activation could limit the anti-mitogenic effect of PTHrP and cAMP in H1944 cells. However, the ERK proliferative effect is small, so ERK phosphorylation would be a weak mitogenic stimulus at best in H1944 cells. The greatest effects of ERK knockdown occurred in PTHrP-positive cells, the cells where ERK phosphorylation was greatest.

Interpretation of the ERK1 knockdown experiments was complicated by a concomitant substantial increase in ERK2 expression. The increase in growth after ERK1 knockdown could be most simply ascribed to a pro-proliferative effect from the increase in ERK2. However, we cannot rule out the possibility that ERK1 activation inhibits growth and that reduction in its activity had a direct effect to increase cell proliferation in addition to the indirect effect on ERK2. ERK1 knockdown did not have a substantive effect on the growth inhibitory action of the cAMP analogs, consistent with the proposal for a minimal role in regulating H1944 proliferation. The change in ERK2 levels with ERK1 knockdown was unexpected and suggests that the expression of one ERK isoform may regulate the other in lung cancer cells. The finding is novel as we know of no previous report of such a feedback arrangement.

## Conclusions

In summary, this study explored two aspects of the upstream signaling mechanisms through which PTHrP regulates lung cancer cell proliferation. The first finding was that cell surface interactions of PTHrP 1–34 with PTH1R inhibit proliferation. The effect may be mediated, in part, by cAMP as a second messenger. The second major finding was that activation of ERK could not account for PTHrP-mediated growth inhibition. Instead, ERK2 phosphorylation led to modest stimulation of proliferation, while the effect of activated ERK1 was poorly defined. Thus, PTHrP-mediated growth inhibition would be somewhat larger if not for the action of ERK2. The finding that PTHrP interferes with lung cancer cell growth by binding to its cell-surface receptor, PTH1R, could have clinical implications. It is conceivable that synthetic ligands or small molecule drugs could be designed to activate PTH1R and inhibit lung carcinoma growth.
